# Personal Observations and Cases

**Published:** 1856-02

**Authors:** 


*Personal Observations and Cases.*

We thought that we had been constantly giving in the pages of our *Journal* more of our own practice and observations than was either customary or modest. But a subscriber writes, requesting us to give *more* of the cases, medical and surgical, treated in the hospital, &c.—Desirous of acceding to every reasonable request, we give the following:—

CASE I.—*Warty Excrescences of the Labia:*—Miss A———, a native of France, aged about 20 years, was admitted into the female ward of the Mercy Hospital, in this city, early in October last. On examination I found her in apparently good health, with no evidence that I could find of previous syphilitic disease. But the whole exterior surface of both labia, was covered with a large mass of warty excrescences, presenting a pale red color, not sensitive to the touch, nor in any degree painful.

The bulk of the excrescences, together with the roughness, chafed the neighboring parts whenever the patient walked about, which kept up a slight erythematic redness of the vulva, with a little serous discharge. Besides the principal masses of warts on the labia, isolated ones projected from the skin, as far out as the upper and inner part of the thighs, and varying in size from a quarter to half an inch in length, with small pedicles. As the larger masses of excrescences could not be readily destroyed by caustics, I armed two or three suture needles with ligatures, and passing them through the base of the morbid growth, tied them in such directions as to include the greater part. Severe pain followed the tying of the ligatures, which was allayed somewhat by keeping the parts wet with a solution of morphine. At the end of three or four days much of the morbid growth included in the ligatures, had become dark colored, shriveled, and very offensive. I now took the scalpel and forceps, and not only finished the separation of the parts included in the ligatures, but also excised nearly all the more scattering or isolated excrescences. This gave rise to a pretty free hemorrhage; several arteries being large enough to throw a pulsating stream from the cut surface.

This was suppressed, however, without the use of ligatures, by applying freely pulverized mattics, aided by pressure with a large sponge for ten or fifteen minutes. During the next three days the parts were simply kept clean, and once a day wet with a solution of sulphate of iron ℈j. to the pint of water. The surface, wherever the warty growth had been completely removed, cicatrized rapidly and became covered with healthy skin; but in some places a little of the morbid growth remained, and the daily application of solution of sulphate of iron or copper served merely to prevent the renewal of its growth without causing it to disappear. I then directed an ointment, consisting of pulv. savine, 20 grs., acetate of copper, 20 grs., and simple cerate, ℥j., to be applied to the diseased parts once a day. Under the use of this she recovered completely, and was discharged at the end of the sixth week after her enterance into the hospital. During a part of the time she took iodide of potassa in doses of 5 grs., three times a day, with an occasional laxative to open the bowels. Two months have elapsed since her discharge and she remains well.

---

Case II.—Mr. J. H., a German, aged about 25 years, was admitted to the medical wards of the Mercy Hospital Jan. 25th, 1856. He stated that he had been suffering more or less from intermittent fever, during the previous summer and autumn; that six weeks since he was attacked with acute pain in the right sub-auxiliary region, accompanied by fever, short cough, difficulty of breathing, &c. After a few days the dyspnœa became so much increased that he could not lie in a horizontal position, and the sense of fullness and oppression in the right side of the chest became very great. In about three weeks after the attack of pain in the side, the fever and all acute symptoms had much diminished, except the sense of fullness in the side and the dyspnœa. About that time the patient noticed a distinct swelling on the right side over the sixth and seventh ribs and directly below the axilla. This swelling continued slowly to increase, until his admission into the hospital, where he first came under my personal observation.

On examining him carefully before the class assembled for clinical instruction, I found his lips pale, skin sallow, considerable emaciation, pulse 100 per minute and small, tongue clean, but little thirst, bowels nearly natural, urine rather scanty, and high colored, inspiration short, with much greater motion of the left side of the chest than the right; some pain in the right side, increased by coughing or an effort to take a full inspiration; constant feeling of fulness in the right side of the chest, which is much increased by the horizontal position. Directly below the right axilla and over the fifth, sixth, and seventh ribs, there was a prominent swelling, moderately tender to the touch, and feeling firm and hard, except on its most prominent part over the spaces between the sixth and seventh ribs, where it was more yielding and elastic, but without distinct fluctuation. The intercostal spaces from the fourth rib downwards were full and bulging on the right side, but moderately depressed on the left. Percussion elicited a dull sound over the mammary, axillary, and sub-axillary regions of the right side, while over the left side the resonance was normal. No respiratory sound could be detected by auscultation in any part of the right side below the line of the fourth rib. Above that line and throughout the left side respiratory murmur was plain and very nearly normal. The patient had very little cough or expectoration, though there was a visibly increased febrile movement every afternoon and evening—and he could rest comfortably only on his back, with the head and shoulders elevated. The first question of importance in this case related to the *diagnosis*. Did the swelling of the right side originate from disease of the thoracic parieties, of the upper convex surface of the liver, or of the pleura.

The absence of signs of enlargement of the liver downward beneath the margin of the ribs; the absence of tenderness from percussion over its central portion; and the evidence of a healthy secretion in the intestinal evacuations, constitute, at least, negative evidence that the liver is not involved in the disease. On the other hand, the dulness on percussion over the whole lower part of the right side of the chest; the absence of respiratory sounds over the same region; the fulness of the intercostal spaces and enlargement of that side; together with the nature of the pain at the commencement of the attack, and the effect on the act of respiration, afford very positive evidence that the swelling is connected with something which has distended the pleura and compressed the right lung. There could be, therefore, but little hesitation in regarding the case as one of empyema, resulting from an attack of sub-acute pleurisy. To be further assured, however, concerning the contents of the tumor or external swelling, I punctured it with a grooved exploring needle. When the needle had entered about one inch, the resistance suddenly ceased as though it had entered a cavity; and on giving it a slight rotary motion two or three drops of pus escaped through the groove. After withdrawing the needle, I passed a small probe through the puncture, which first struck the upper margin of the rib; but on changing its direction a little, it passed readily into the chest, without resistance its whole length. I withdrew the probe, and ordered a poultice of linseed meal to the side, intending, the next day, to request the attending surgeon, Professor Brainard, to perform the operation of paracentisis thoracis in the usual manner. To lessen the general irritative fever, and remove the remaining inflammatory action in the pleura, I directed internally, a powder composed of pulv. doveri, 5 grs., sub-murias hydrarg. 2 grs., pulv. digitalis, 1 gr., to be given every six hours. On removing the poultice next day, I found that the small opening previously made by the exploring needle and the probe, had permitted a very slow but constant discharge of pus. Knowing that in many cases of this kind, the lung in its compressed state becomes bound down by adhesions so firm as to prevent its immediate expansion when the pus or fluid is evacuated, thereby leading to ultimate contraction of the affected side, it occurred to me that the present slow and constant discharge through the small opening would be more likely to give the lung time to expand in proportion as the fluid was evacuated, than if it were drawn off more rapidly by the usual operation.

Hence emollient poultices were continued on the side, and the same internal treatment continued. At the end of two days the small amount of mercurial medicine in his powders had began to effect his gums and salivary glands. The powders were discontinued, and in their place I directed the iodide of potassa, 5 grs., four times a day, and a mild nutritious diet.

In the meantime, the discharge of pus continued from the puncture in the side, amounting in quantity from two to four ounces in 24 hours. The sense of fulness in that side of the chest, and the difficulty of lying down in the recumbent posture, were diminished. The pulse also became slow, and the slight febrile irritation disappeared. He did not take the iodide of potassa more than three days before his bowels became loose, with frequent griping pains in the abdomen, requiring its omission and the use of anodynes in its place.

It is now about three weeks since the patient came under treatment, and the puncture was made in the tumor. The discharge of pus continues, but in very much smaller quantity and of a more healthy appearance; the right side has diminished in size; the intercostal spaces are less full; the dulness on percussion is less; and the respiratory murmur, though feeble, can be heard below the nipple and in the axilla. There are no febrile symptoms and no pains in the chest. Still the patient is debilitated, with some tendency to diarrhœa. Sufficient improvement has been made, however, to leave no doubt about his ultimate recovery.

CASE III.—*Direct Inguinal Hernia, Operation, &c.*—Jan. 26th, 1856; was called to visit Mrs. W———, aged about 35 years. Found her affected with severe pain in the abdomen, of a paroxysmal character, coupled with constipation of the bowels, some nausea, and slight general febrile action. The patient also complained of some tenderness in the right iliac region. She said she had been subject to such turns occasionally for several years; and attributed the present attack to the influence of cold and a long walk the day previous. Regarding the severe pains in the abdomen as spasmodic and dependent on some slight irritation in the intestines, I directed the following:

| | | |
|---|---|---|
| ℞. | Sulph. Morph., | 2 grs. |
| | Sub. Murias Hydrarg., | 10 " |
| | White Sugar, | 20 " |

Mix, divide into four powders; one to be given every three hours, with fomentations over the abdomen. After the powders had been taken and the severe pain relieved, she was to take rochelle salts, in the form of effervescing draughts, until free evacuations were induced.

On the 27th, the pain was much less and the patient comparatively comfortable, though complaining of some nausea, and tenderness of the abdomen; but she had no movement of the bowels.

She also mentioned the existence of a swelling in the "right groin," which she said had existed six or seven years; but as she said it was hard, located in the groin, and had never receded or disappeared by pressure, or taking the recumbent posture, I did not insist on a direct examination, thinking it might be only an enlarged lymphatic gland. As she was suffering much less pain, and had rejected a part of the Rochelle salts, I directed the stomach to be left at rest, the fomentations to be continued over the abdomen, and the bowels to be moved, if possible, by laxative enemas. She followed these directions during this and the succeeding day, but with no other effect than the procurement of one or two slight fœcal discharges, not more, however, than would naturally have been retained in the lower part of the colon and rectum. I found her slightly everish, inclined to vomit up every thing she drank, considerable pain in the unbilical and right iliac regions, of a paroxysmal character, and still some tenderness of the abdomen.

I now deemed it prudent to examine carefully the tumor before mentioned as being in the groin. I found it occupying the right inguinal region; about four inches in its longest diameter; of a semi-elastic feel, and moderately tender to the touch.

It occupied the space directly in front and below the external inguinal ring, and was doubtless an irreducible inguinal hernia, of six or seven years duration. As the tumor was moderately tender, and had never been reduced since it first made its appearance, I did not deem it prudent to make more than moderate efforts at reduction by taxis. As the patient claimed to have been several times in the same condition in regard to the bowels, and yet by persevering efforts succeeded in obtaining relief by ordinary means, I was induced to delay recommending a resort to the usual surgical operation. To allay the severe paroxysmal pains in the abdomen, and at the same time powerfully dispose the bowels to move, I directed a weak infusion of tobacco with senna, to be used as an enema in sufficient quantity to distend the rectum; and to be repeated every two or three hours, if no undue prostration was induced by the tobacco. The stomach was too irritable to retain either medicine or drinks. By the cautious use of the tobacco enemas, the severe paroxysms of pain, together with the symptoms of inflammation, were kept so far under control as to prevent the development of any alarming symptoms for three days. On the morning of the 2nd of February, one week from the time she was taken sick, she expressed herself as feeling weak, and nauseated, but more as though she should have an evacuation from the bowels, than at any time previous.

As there was still not much tympanitic distention of the abdomen, nor indications of inflammation, I ventured to administer a single dose of active cathartic medicine, to be followed in two hours by another tobacco enema of sufficient strength to produce decided relaxation of the system.—At evening of the same day, I found the patient had rejected the cathartic administered in the morning; and instead of procuring an evacuation from the bowels, it had much increased the pain in the abdomen, accompanied by constant nausea, with a distressing sense of heat in the stomach; a quicker pulse, and more tenderness both in the hernial tumor and the neighboring region. Being satisfied that any further attempts to procure foecal discharges, without first removing the stricture from the neck of the hernial sac, would be dangerous to the patient, I directed half a grain of morphine to be given at intervals of two or three hours to allay pain and procure rest during the night; and sent a request to my colleague, Professor BRAINARD, to meet me prepared to operate for strangulated hernia, the following morning at eight o'clock. At the hour appointed we found the patient quiet, had slept a little during the night, her abdomen not tympanitic and only slightly tender to pressure, the pulse 100 per minute and soft, the skin of the natural temperature, but the stomach too irritable to retain either drinks or nourishment. It had now been one week since any foecal discharges had been procured. Prof. BRAINARD fully coinciding with me in regard to the existence of a strangulated hernia, and the necessity for obtaining speedy relief, he proceeded at once to the usual operation. After a few minutes inhalation the patient passed pretty fully under the influence of ether, and he made an incision through the skin in the direction of the longitudinal diameter of the tumor. The several layers of cellular tissue and facia were raised on the grooved director and laid open, bringing the peritoneal portion of the sac fully into view. It appeared somewhat ecchymosed, and was quite irregular in shape; one lobe extending downward towards Poupart's ligament, and another downward and inward towards the Labia Pudendum. On puncturing the sac, and laying it open over the director, a little serous fluid escaped, but the larger part of its contents was found to be omentum, not unnatural in its appearance, but of a temperature considerably less than the other structures. Accompanying the omentum was a loop of intestine distended with foeces, but without any decided appearances of either inflammation or gangrene. By a careful examination, Prof. BRAINARD found the hernia to have been *direct*, with the artery passing close along the outer margin of the opening. He consequently made the incision necessary for enlarging the opening, in the direction upwards and inwards, and returned both the loop of intestine and the protruding portion of omentum into the abdomen. The external wound was then closed with three sutenes or stitepes, the middle one of which was inserted deep enough to include the inner edges of the opening. The anesthetic effects of the ether passed away quickly, and the operation was found to have produced but a slight degree of general depression. As no nourishment had been retained on the stomach during the last six days, she was directed to take beef-tea in doses of one table-spoonful every half hour, and if no discharge from the bowels occurred before the end of four hours, an enema of warm water was to be administered. In a few hours, foecal discharges occurred, but only moderate in quantity, accompanied by little pain. The nausea and vomiting also ceased, and the patient continued to progress favorably until the morning of the third day. She was then found to be restless, feverish, very thirsty, pulse 110 per minute, abdomen moderately distended, with a great feeling of tenderness and sense of tension in the right inguinal region. There was little or no discharge from the wound, the edges of which were somewhat swollen and tender. Apprehending the rapid development of peritoneal inflammation, I directed the following:

| | |
|---|---|
| ℞. Sub. Murias., Hydrarg., | 12 grs. |
| Sulph. Morph., | 2 grs. |
| Bi Carb. Soda, | 20 grs. |

Mix, divide into 4 powders; one to be taken every two hours, and to be followed by an enema of castor oil, with oil of turpentine.

During the day, Professor Brainard called and cut the sutures, which considerably relieved the sense of tension and pain in the part. On the following day, the fourth after the operation, the symptoms were again much improved. The patient was less restless, less fulness and pain in the abdomen, pulse only 90 per minute, and the bowels had been moved during the preceding night freely, the foecal discharges being dark colored, lumpy, and offensive.

There was also considerable purulent discharge from the external wound. She was troubled with a slight, but annoying cough, for which I directed a mixture of compound honey of squills one part and camphorated tinct. of opium three parts, a tea-spoonful every three or four hours. The stomach remained quiet and she continued to take animal broth for nourishment.

During the fifth and sixth days after the operation, the discharge of matter from the external wound was very much increased, and extremely offensive to the smell. The pus was thin, of a dark brown color, and copiously intermixed with large globules of oil.

On the sixth day several tough pieces of gangrenous tissue presented themselves at the external opening, and were partially removed by Prof. BRAINARD. The remainder of these were discharged with the offensive matter during the following night.

During these two days the discharges from the bowels were too frequent and thin, but not otherwise unhealthy, and her general symptoms remained favorable. To lessen the offensiveness of the purulent discharge, a wash of liquid chloride of soda was directed, and the bowels were restrained by an emulsion of oil of turpentine and tinct. of opium. The pieces of gangrenous tissue discharged were evidently a part of the omentum which had been previously strangulated in the hernial sac. On the eighth day after the operation, the purulent discharge was less copious and offensive, the bowels were less disposed to move frequently, and the patient felt much more comfortable. From this time the discharge daily diminished, the bowels became more regular, the appetite became good; and at this time, Feb. 22d, she is nearly well.

I trust these cases will satisfy our subscriber for this time.

